# Increased expression of Foxp3 in splenic CD8^+^ T cells from mice with anterior chamber-associated immune deviation

**Published:** 2007-06-19

**Authors:** Liqiong Jiang, Peizeng Yang, Hao He, Bing Li, Xiaomin Lin, Shengping Hou, Hongyan Zhou, Xiangkun Huang, Aize Kijlstra

**Affiliations:** 1 State Key laboratory of Ophthalmology, Zhongshan Ophthalmic Center, Uveitis Study Center, Sun Yat-sen University, Guangzhou, China; 2 Eye Research Institute, Department of Ophthalmology, Maastricht, Netherlands

## Abstract

**Purpose:**

To examine the expression of Foxp3 on CD8^+^ T cells in the spleen during anterior chamber-associated immune deviation (ACAID).

**Methods:**

Ovalbumin (OVA) was injected into the anterior chamber (AC) of C57BL/6 mice, and the delayed-type hypersensitivity (DTH) response was measured to evaluate the development of ACAID. The suppressive effect of CD8^+^ T cells in ACAID mice was determined by a local adoptive transfer (LAT) assay. Flow cytometry was used to assay the frequency of CD8^+^Foxp3^+^ T cells from normal mice, ACAID mice, and control mice receiving an AC injection of PBS (PBS-AC-injected mice). These frequencies were also tested after polyclonal or specific antigen stimulation. The mRNA level of Foxp3 in CD8^+^ splenocytes from different groups with or without stimulation were determined by reverse transcription-polymerase chain reaction.

**Results:**

OVA injection into the AC induced ACAID, and CD8^+^ T cells from ACAID mice inhibited the ear-swelling response by OVA-primed responder cells in LAT assay. Flow cytometry analysis showed that the frequency of CD8^+^Foxp3^+^ cells in splenocytes was upregulated in ACAID mice following polyclonal or specific antigen stimulation. Foxp3 mRNA was only detected in CD8^+^ T cells from ACAID mice after polyclonal stimulation.

**Conclusions:**

An upregulated Foxp3 expression in CD8^+^ T cells is associated with the development of ACAID, suggesting an involvement of CD8^+^Foxp3^+^ T cells in this model of immune tolerance.

## Introduction

A variety of antigens, including soluble proteins, viral-encoded proteins, and minor histocompatibility antigens, elicit a deviant systemic immune response when injected into the anterior chamber (AC) of the eye. This immune deviation is termed anterior chamber-associated immune deviation (ACAID) [1,2]. It is characterized by impaired Ag-specific delayed-type hypersensitivity (DTH), expanded clones of precursor cytotoxic T cells, and elevated serum levels of noncomplement-fixing Abs [3]. During the induction of ACAID, intraocular antigen-presenting cells capture the antigen placed in the eye and carry it to the marginal zone of the spleen. In the presence of thymus-derived natural killer T cells and marginal zone B cells, a microenvironment capable of activating antigen-specific T cells to differentiate into regulatory cells is created in the spleen. A number of studies have demonstrated that there are two distinct populations of regulatory T cells (Tregs) involved in the suppression of DTH responses in ACAID [3-9]. One population comprises CD4^+^ T cells, which block the induction or afferent component of the immune response. Another population consists of CD8^+^ T cells, which inhibit the expression of DTH through previously sensitized T cells.

The crucial role of Tregs in the maintenance of immune tolerance and in the control of autoimmunity has been intensively investigated during the last decades. Up to now, several markers, such as CD25 [10], GITR [11], CTLA-4 [12], and LAG-3 [13], have been used to define Tregs. However, they are also associated with the activation of T cells. Foxp3, a kind of forkhead transcription factor encoded by the X chromosome, has been considered a specific marker of Treg lineage [14-20]. It is not upregulated in recently activated CD4^+^CD25^-^ T cells. Mice with a deficiency in Foxp3 show a rapid, fatal lymphoproliferative autoimmune syndrome [16,20]. Ectopic expression of Foxp3 can confer suppressor activity on some nonregulatory T cells [15-17]. Since the great majority of Foxp3-expressing cells are CD4^+^ T cells [14], most studies have addressed the role of CD4^+^ Tregs in immune tolerance. More recently, the role of CD8^+^ Tregs during immune tolerance has also been emphasized [21]. Increasing evidence has indicated that CD8^+^ regulatory T cells can express Foxp3 in both humans and rodents [21-24]. The expression of Foxp3 on CD8^+^ T cells during ACAID is not well known and was, therefore, the subject of our study. Here we show that splenic CD8^+^ T cells from ACAID mice express Foxp3 after polyclonal and specific antigen stimulation.

## Methods

### Mice

Specific pathogen-free female C57BL/6 (B6; H-2^b^), 6-8-week-old mice were purchased from the animal facility at the Sun Yat-Sen University, P.R. China. All mice were treated according to the ARVO Statement for the Use of Animals in Ophthalmic and Vision Research. The numbers of mice used in the various experiments are described below.

### Reagents

OVA was purchased from Sigma-Aldrich (Steinheim, Germany) and dissolved in phosphate-buffered saline (PBS). Complete Freund's adjuvant (CFA) containing heat-killed *Mycobacterium tuberculosis* strain H37Ra was purchased from Sigma-Aldrich. The Abs used in flow cytometry (FCM) were Fc blocker (affinity purified anti-mouse CD16/32, clone 93), fluorescein isothiocyanate (FITC)-conjugated antimouse CD3e (clone 145-2C11), allophycocyanin (APC)-conjugated antimouse/antirat Foxp3 staining set (clone FJK-16s), and APC-conjugated rat IgG2a isotype control (all purchased from eBioscience, San Diego, CA). Phycoerythrin-Cy5.5 (PE-Cy5.5)-conjugated antimouse CD8a (clone 5H10) was purchased from Caltag Laboratories (Burlingame, CA). FITC-conjugated antimouse F4/80 antigen (clone CI:A3) was purchased from Serotec (Oxford, UK).

### Treatment of mice

ACAID was induced as described previously using microinjection of antigen into the AC of the eye [25]. Briefly, mice were anesthetized and 5 μl OVA (20 mg/ml) was injected into the AC by a glass micropipette with a sterile infant feeding tube mounted onto a 0.1 ml Hamilton (Hamilton, Reno, NV) syringe. Mice receiving an AC-injection of PBS served as a control (PBS-AC-injected mice). Primed mice received a subcutaneous (SC) injection of 250 μg of OVA in PBS. To induce a DTH response, we gave the mice soluble antigen emulsified 1:1 in CFA. Each animal received a total volume of 200 μl antigen in CFA.

### Impaired Ag-specific delayed-type hypersensitivity assay

The ear-swelling response was measured to indicate DTH response to OVA as described previously [26-28]. Both ear pinnae of ACAID mice and control animals were measured with a Mitutoyo engineer's micrometer (MTI Corp., Paramus, NJ) immediately before challenge. In these experiments, primed mice served as positive controls and untreated mice served as negative controls. OVA (400 μg) in 20 μl PBS was injected SC into the right ear pinnae. The left ear pinnae received 20 μl sterile PBS alone. Both ears were measured 24 h later, and the difference in thickness was used as a measurement of DTH. Results were expressed as follows: specific ear swelling (24 h measurement-0 h measurement) of the right ear (24 h measurement-0 h measurement) of the left ear. Five mice were used for each group through one experiment and the experiment was repeated twice.

### Preparation of peritoneal exudate cells

Peritoneal exudates cells (PECs) were harvested from C57BL/6 mice that had received 2.5 ml of thioglycolate (Sigma-Aldrich) intraperitoneally three days earlier. As described previously [29], the recovered cells were washed and resuspended, placed in 24-well culture plates (1x10^6^ cells/well), and incubated in complete RPMI 1640 medium at 37 °C in an atmosphere of 5% CO_2_. After overnight culture, plates were washed three times with culture medium to remove nonadherent cells. Adherent cells were retained in the wells and used as antigen-presenting cells in subsequent experiments. To test the purity of the adherent cells through FCM, we harvested these cells after incubation on ice for 1 h and then gently removed them with a cell-lifter. More than 90% of the adherent cells were identified as being F4/80^+^ cells.

### Cell isolation and culture

Fourteen days after AC injection, single cell suspensions of splenocytes were prepared by passing cells through a sterile wire mesh. Red blood cells in spleens were lysed with Tris-NH_4_Cl or removed through density gradient centrifugation. In some experiments, the total splenocyte population was cultured in complete RPMI 1640 medium with stimulation of anti-CD3 and anti-CD28 mAb or with OVA for 36 h and used for FCM analysis of Foxp3 expression. These splenocytes were cultured in a density of 2x10^6^ cells/ml in 24-well culture plates. The volume was 1 ml for each well. CD8^+^ T cells were isolated from splenocytes, using magnetic separation columns (MiniMACS, MS columns; Miltenyi Biotec, Bergisch-Gladbach, Germany) according to the manufacturer's instructions. After positive selection, the purity of CD8^+^ T cells was >95% according to subsequent FCM analysis. The purified CD8^+^ T cells were then cultured in 24-well culture plates (2x10^6^ cells/well) containing isolated PECs with 200 μg/ml of OVA at 37 °C in complete RPMI 1640 medium for 36 h and used for RT-PCR analysis of Foxp3 expression.

### Local adoptive transfer assay

A local adoptive transfer (LAT) assay was developed to test suppressor cells during ACAID [30,31]. Putative suppressor cells consisting of purified CD8^+^ T cells were separated from splenocytes of AC-injected mice. Immune cells were collected from splenocytes of primed mice. Both suppressor cells and immune cells were harvested on day 14 after injection and were suspended at 5x10^7^ cells/ml in 10 mg/ml OVA in PBS. The immune and suppressor cells populations were then mixed 1:1 in the presence of antigen (10 mg/ml). Both ears of naïve C57BL/6 mice were measured with an engineer's micrometer immediately before challenge. The right ear pinnae of naïve C57BL/6 mice were injected with 20 μl (1x10^6^ cells) of the mixed-cell population. The left ear pinnae were injected with 10 mg/ml of OVA in PBS (negative control). Ear swelling was measured 24 h later to evaluate DTH. Naïve T cells from nonmanipulated mice were used as immune cells and purified CD8^+^ T cells from these mice were used as suppressor cells for negative controls. Primed T cells were used as immune cells, and CD8^+^ T cells were purified from nonmanipulated mice and used as suppressor cells for positive controls. In one experiment, two ACAID mice and three normal mice were used to generate putative suppressor cells, and one primed mouse was used to generate immune cells. Five naïve mice were used for measuring the ear-swelling response for each group in one experiment. The experiment was repeated twice.

### Flow cytometry

Analysis of Foxp3 protein expression in stimulated or unstimulated splenocytes from normal, ACAID, and PBS-AC-injected mice was performed with BD FACSAria (BD Biosciences, San Jose, CA) using the BD FACSDiVa software (BD Biosciences). Cells were prepared according to the manufacturer's instructions using the antimouse/rat Foxp3 staining set. Briefly, 1x10^6^ cells were first stained with FITC-CD3e mAb and Pecy5.5-CD8a mAb at 4 °C for 30 min, then fixed and permeabilized using fixation/permeabilization buffer at 4 °C for 2 h. Cells were washed in permeabilization buffer and then incubated with Fc blocker for 15 min. The cells were not washed prior to being incubated with APC-Foxp3 at 4 °C for another 30 min. After washing, the cells were resuspended and subjected to FCM. Cells stained with APC-conjugated rat IgG2a served as isotype controls. In vitro, the cells stimulated with BSA served as irrelevant antigen controls. Four mice were used for each group in one experiment. These experiments were repeated twice.

### Reverse transcription-PCR

Total cellular RNA was isolated from 5x10^6^ purified CD8^+^ cells with or without stimulation using Trizol (Invitrogen, San Diego, CA). The total amount of RNA was reverse transcribed using Superscript III reverse-transcriptase (Invitrogen) and oligo(dT) primer (Tiangen, Beijing, China) in a final volume of 20 μl. First strand cDNA (1.0 μl) was amplified in a 25 μl reaction using Taq Platinum PCR MasterMix (Tiangen). β-Actin was used as a control. For Foxp3 amplification, PCR reactions consisted of a 3 min denaturation step at 94 °C followed by 40 cycles of 30 s at 94 °C, 1 min at 48 °C, and 30 s at 72 °C. For β-actin, reactions were conducted as described above except that the annealing temperature was 54 °C for 30 s and the number of cycles was 30. The following primers were used: Foxp3-FW, 5'-CCC AGG AAA GAC AGC AAC CTT-3'; Foxp3-RV, 5'-CCT TGC CTT TCT CAT CCA GGA-3'; β-actin-FW, 5'-GTC CCT CAC CCT CCC AAA AG-3'; and β-actin-RV, 5'-GCT GCC TCA ACA CCT CAA CCC-3'. Spleen cells from two mice were used in each group in one experiment. The experiment was repeated twice.

### Statistics

Statistical analyses were performed by one-way ANOVA using SPSS 11.0. A p<0.05 was considered significant.

## Results

### Local adoptively transferred CD8^+^ T cells from anterior chamber-associated immune deviation mice are able to inhibit the Ag-specific delayed-type hypersensitivity response in naïve recipients

**Figure 1:**
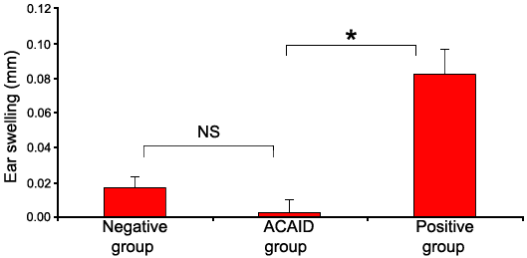
Effect of CD8^+^ T cells from anterior chamber-associated immune deviation mice on the expression of impaired Ag-specific delayed type hypersensitivity in vivo. Purified CD8^+^ T cells from splenocytes were used as regulator cells. Regulator cells and ovalbumin (OVA)-primed responder cells were prepared 14 days after antigen injection. The regulator cells and responder cells were mixed 1:1 in the presence of OVA and transferred to the ear pinnae of naïve C57BL/6 mice. As a negative control, naïve spleen cells were used as responder cells and purified CD8^+^ T cells from untreated mice were used as regulatory cells. Primed spleen cells were used as responder cells, and purified CD8^+^ T cells from untreated mice were used as regulatory cells for a positive control. Ear swelling was measured at 24 h. Mean±SEM ear-swelling responses are presented (n=5). The asterisk indicates a p<0.05, and NS denotes a p>0.05. The experiments were repeated twice with similar results.

As reported by others [26] and documented in our group's earlier work [27,28], ACAID was successfully induced by AC injection of OVA. The LAT assay was used to study the inhibitory function of antigen-specific efferent suppressor cells in ACAID. In this experiment, CD8^+^ T cells were purified, mixed with responder cells and finally transferred in the presence of OVA to the ear pinnae of naïve animals. The ear-swelling responses of mice that received responder cells mixed with CD8^+^ cells from normal mice displayed significant ear swelling indicative of a DTH response (positive control). However, this ear-swelling response induced by responder cells was significantly reduced when CD8^+^ cells from ACAID mice were coinjected with these cells. The result of a typical experiment is shown in Figure 1.

### The frequency of CD8^+^ T cells is increased in the spleen of anterior chamber-associated immune deviation mice

**Figure 2:**
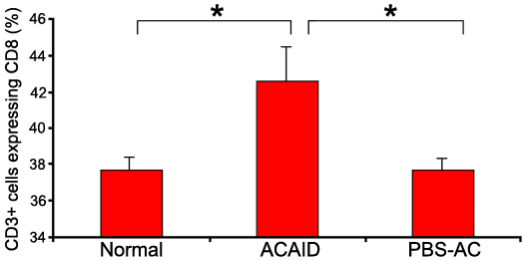
Flow cytometry analysis of CD8^+^ T cells in different groups. Anterior chamber-associated immune deviation (ACAID) group received an anterior chamber (AC) injection of ovalbumin (OVA). The phosphate-buffered saline-anterior chamber (PBS-AC) group received an AC injection of PBS. We gated on CD3^+^ T cells, and the frequency of CD8^+^ T cells is shown on the vertical axis. Results represent the mean±SEM of two separate experiments. The asterisk indicates a p<0.05.

FCM was used to examine the frequency of CD8^+^ T cells in splenocytes harvested 14 days after an AC injection of OVA. The results showed that the frequency of CD8^+^ T cells from the spleen of ACAID mice was 42.5% of the CD3^+^ gated splenocyte population, which was significantly higher than that from normal mice (37.7%, p=0.043) and PBS-AC-injected mice (37.8%, p=0.034; Figure 2). There was no significant difference between the normal mice and the PBS-AC-injected mice (p=0.969).

### CD8^+^ cells from anterior chamber-associated immune deviation mice express increased Foxp3 protein upon activation

**Figure 3:**
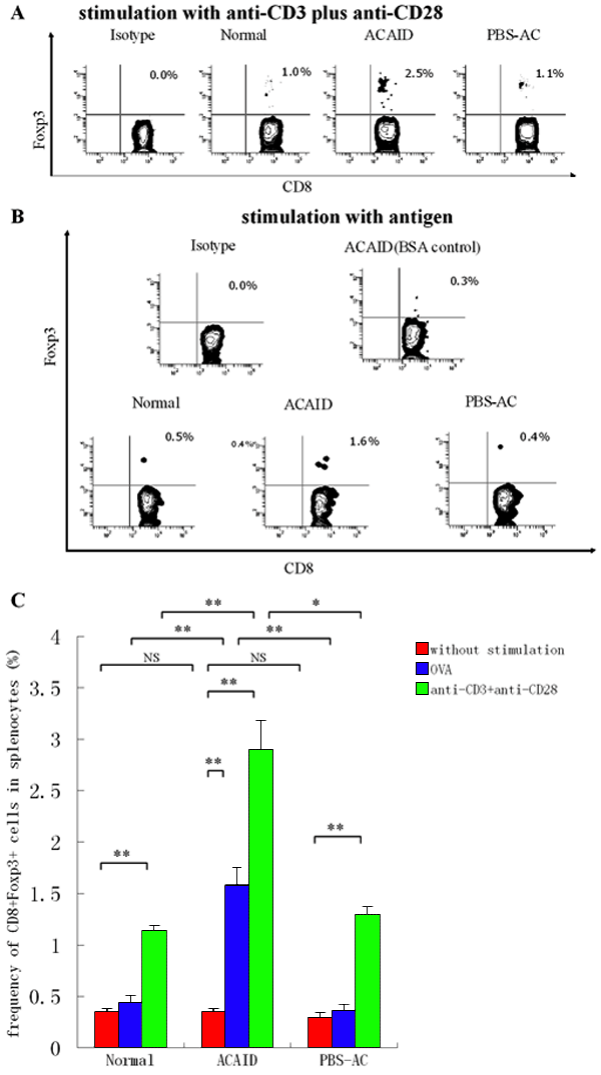
Flow cytometry analysis of Foxp3 protein expression in CD8^+^ T cells from normal mice, anterior chamber-associated immune deviation mice, and phosphate-buffered saline-anterior chamber-injected mice without stimulation or with specific or nonspecific stimulation. Whole spleen cells were obtained from C57BL/6 mice 14 days after anterior chamber (AC) injection. Intracellular staining of Foxp3 is shown in gated CD8^+^ cells from anterior chamber-associated immune deviation (ACAID) mice, phosphate-buffered saline-anterior chamber (PBS-AC)-injected mice and normal mice. In the ACAID group, the mice received an AC injection of 5 μl ovalbumin (OVA; 20 mg/ml). PBS-AC-injected mice received an AC injection of 5 μl PBS. **A**: Representative data showing the expression of Foxp3 on CD8^+^ splenocytes from normal, ACAID, and PBS-AC-injected mice following polyclonal stimulation. **B**: Representative data showing the expression of Foxp3 on CD8^+^ splenocytes from normal, ACAID, and PBS-AC-injected mice after stimulation with OVA or BSA control. **C**: The statistic histogram for the frequencies of CD8^+^Foxp3^+^ cells in different groups with or without stimulation. Results represent the mean±SEM of two separate experiments. The double asterisk indicates a p<0.01, and NS denotes a p>0.05.

FCM was employed to examine Foxp3 expression in CD8^+^ splenocytes during ACAID production. As shown in Figure 3, the frequency of CD8^+^Foxp3^+^ T cells was 0.35%, 0.35%, and 0.30%, respectively, in the CD8^+^ splenocytes from ACAID mice, normal mice, and PBS-AC-injected mice, none of whom received stimulation. There were no significant differences among these three groups. We further assayed the frequency of CD8^+^Foxp3^+^ T cells in different groups after in vitro stimulation either with anti-CD3 plus anti-CD28 or with OVA (Figure 3). The results showed that the frequency of CD8^+^Foxp3^+^ T cells in the spleen of normal mice was significantly increased after polyclonal stimulation (0.35% versus 1.14%; p<0.001), but only slightly after OVA stimulation (0.35% versus 0.44%; p=0.297). A similar result was also observed in the spleen of PBS-AC-injected mice (0.30% versus 1.30%; p<0.001 in polyclonal stimulation; 0.30% versus 0.36%; p=0.436 in OVA stimulation). The frequencies of CD8^+^Foxp3^+^ T cells were both increased significantly in ACAID mice after stimulation with either anti-CD3 plus anti-CD28 mAb (0.35% versus 2.90%; p<0.001) or OVA (0.35% versus 1.58%; p<0.001) in vitro. The frequency of CD8^+^Foxp3^+^ T cells were significantly higher in ACAID mice than that in normal mice (2.90% versus 1.14%; p=0.009 in non-specific stimulation; 1.58% versus 0.44%; p=0.005 in specific stimulation) and in PBS-AC-injected mice after both stimulations (2.90% versus 1.30%; p=0.012 in nonspecific stimulation; 1.58% versus 0.36%; p=0.004 in specific stimulation).

### Expression of Foxp3 mRNA is found in CD8^+^ T cells from anterior chamber-associated immune deviation mice after polyclonal stimulation

**Figure 4:**
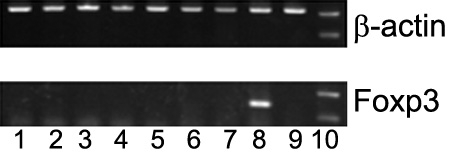
Reverse transcription-PCR analysis of Foxp3 expression in CD8^+^ T cells from normal mice, anterior chamber-associated immune deviation mice, and phosphate-buffered saline-anterior chamber-injected mice without stimulation or with specific or nonspecific stimulation. As a control, β-actin was amplified from the same RNA sample. Lane 1: normal mice without stimulation; lane 2: ACAID mice without stimulation; lane 3: PBS-AC-injected mice without stimulation; lane 4: normal mice stimulated with OVA; lane 5: ACAID mice stimulated with OVA; lane 6: PBS-AC-injected mice stimulated with OVA; lane 7: normal mice stimulated with anti-CD3 plus anti-CD28; lane 8: ACAID mice stimulated with anti-CD3 plus anti-CD28; lane 9: PBS-AC-injected mice stimulated with anti-CD3 plus anti-CD28; lane 10: DNA marker. Results are representative of two independent experiments.

As expression of certain molecules at the protein level and the mRNA level is not always identical, we analyzed the Foxp3 mRNA expression in CD8^+^ splenocytes during ACAID by RT-PCR. As shown in Figure 4, Foxp3 mRNA was not detectable in CD8^+^ T cells from OVA-stimulated and unstimulated ACAID mice, normal mice, or PBS-AC-injected mice. However, the expression of Foxp3 mRNA was noted in CD8^+^ T cells from ACAID mice after stimulation with anti-CD3 plus anti-CD28 mAb. It remained undetectable in CD8^+^ T cells from normal mice and PBS-AC-injected mice. Sequence analysis with the relative primers warranted the RT-PCR products of Foxp3.

## Discussion

In this study, we showed that an injection of OVA into the AC of the murine eye could induce ACAID and that the splenic CD8^+^ T cells from ACAID mice were able to inhibit the DTH response. The expression of Foxp3 protein in the splenic CD8^+^ T cells in ACAID was increased after stimulation with anti-CD3 plus anti-CD28 mAb or specific antigen in vitro. The Foxp3 mRNA was also upregulated in CD8^+^ T cells in ACAID after polyclonal stimulation. These results suggest that expression of Foxp3 may be a property of efferent CD8^+^ Tregs in ACAID.

It has been shown that CD8^+^ Tregs are necessary for inhibiting the expression of DTH in ACAID [5,6]. However, the phenotype of this population remains unclear. For the purpose of studying the phenotype of these Tregs, we first investigated the frequency of CD8^+^ T cells in the spleen. Our study revealed that the frequency of CD8^+^ T cells was increased significantly in the spleen of ACAID mice. This result is consistent not only with expanded clones of precursor cytotoxic T cells [3], one feature of ACAID, but also with the findings presented by McKenna and colleagues [32]. They found that injection of soluble antigen into the AC of the eye induced expansion of antigen-specific CD8^+^ T cells. A further study using a LAT assay was performed to determine the suppressive property of this population. The result showed that CD8^+^ T cells in the spleen of ACAID mice could inhibit the ear swelling of naïve mice produced by primed T cells. This result indicated that efferent suppressor cells were present in the CD8^+^ T cell population residing in the spleen of ACAID mice.

Since Foxp3 has been generally considered to be a specific marker for Tregs both in rodents and humans in spite of CD4 or CD8 lineage [14], a study was performed to examine the expression of Foxp3 in CD8^+^ T cells at both the mRNA level and protein level during ACAID. The results showed that an upregulated expression of Foxp3 in CD8^+^ T cells could only be shown at both the mRNA level and protein level when cells were stimulated using polyclonal activation. These results suggest that a subset of CD8^+^Foxp3^+^ T cells were induced in the ACAID mice and that these T cells were detectable only after stimulation. It was interesting to note that the expression of Foxp3 was up-regulated at the protein level, but not at mRNA level, after specific (OVA) stimulation. A significantly higher expression of Foxp3 protein was also observed with polyclonal stimulation than with specific stimulation. The reasons for a discrepancy between mRNA expression and protein expression after specific stimulation are not fully understood. One explanation is that posttranscriptional control mechanisms may be important for Foxp3 protein expression. This discrepancy was also found in a study by Fontenot et al. [16], who demonstrated that Foxp3 protein expression, but not Foxp3 mRNA expression, was increased following an in vitro activation of naturally existing CD4^+^CD25^+^ Tregs. It is also possible that the findings reflect a difference in sensitivity of the Foxp3 protein versus the mRNA signal. The aforementioned result also suggested that polyclonal stimulation was stronger than specific antigen stimulation in promoting the expression of Foxp3 in both ACAID mice and normal mice using FCM. This result was consistent with the findings by Fontenot et al. [16]. They found the expression of Foxp3 to be associated with the strength of TCR signals during thymic selection.

In order to exclude the influence produced by AC-injection, we performed a study using mice receiving an AC-injection of PBS as a control. The result showed that there was no difference between stimulated and unstimulated PBS-AC-injected control mice and normal mice concerning the expression of Foxp3 in CD8^+^ T cells. This finding suggests that the increased expression of Foxp3 in ACAID is an intrinsic feature of this type of immune tolerance and not a concomitant phenomenon merely induced by AC-injection.

It is interesting to note that the frequency of CD8^+^Foxp3^+^ T cells in ACAID mice was only about 2% even after polyclonal stimulation. This is contrary to a much higher frequency of Foxp3 in CD4^+^CD25^+^ Tregs in ACAID mice (up to 90% in a study in our laboratory [33]). The wild-type mice used in this study may have affected the lower frequency. In these wild-type mice, the great majority of Foxp3-expressing cells are CD4^+^, but not CD8^+^, T cells [14]. A previous study from our laboratory found that the frequency of CD8^+^ cells specific to OVA in wild-type mice was very low [28]. In "OVA T cell receptor" transgenic mice, the frequency of CD8^+^Foxp3^+^ T cells can be expected to be much higher. Sasaki and colleagues [34] found that the expression of Foxp3 was almost 88% in CD8^+^ T cells from such transgenic mice when these T cells were exposed to TGF-β2-treated OVA-pulsed APC in vitro.

Foxp3 expression in CD8+ T cells has also been reported in another immune tolerance model in lupus-prone mice. Hahn et al. [21] injected artificial peptide intravenously into these mice and found induction of Foxp3-expressing CD8^+^ T cells. Functional studies showed that these CD8^+^ T cells suppressed anti-DNA IgG production both in vitro and in vivo. These results, together with ours, suggested that the increased expression of Foxp3 in CD8^+^ Tregs may play a role in the expression of immune tolerance induced either via the AC of the eye or the intravenous route.

Our study has revealed an increased expression of Foxp3 in the CD8^+^ T cells in ACAID mice. To play a role in the inhibition of the DTH response one would expect these cells to express IL-10 and TGF-β. Since the population of these cells is low in ACAID mice, it is currently not yet possible to assay the production of these cytokines. Further resolution of this issue awaits the development of more sensitive techniques. Alternatively, TCR transgenic mice, which have been shown to have numerous CD8^+^Foxp3^+^ T cells, may be suitable for the aforementioned cytokine analysis. A direct role for CD8^+^Foxp3^+^ T in the induction of ACAID has not yet been proven by the data presented by our study. At present we have not succeeded in isolating sufficient numbers of CD8^+^Foxp3^+^ T cells. Other techniques, such as the use of SiRNA for Foxp3, may be used in future experiments to block the function of these cells and definitely prove their role in ACAID.

There are at least two functionally distinct types of Tregs involved in the development of ACAID. They exert a suppressive function during different phases of ACAID. The afferent regulatory cells are CD4^+^, and the efferent regulatory cells are CD8^+^. A recent study by Keino et al. [35] showed the expression of Foxp3 in the CD4^+^CD25^+^ ACAID Tregs. We confirmed these findings and moreover showed that the frequency of CD4^+^Foxp3^+^ T cells was higher in ACAID mice than in normal mice [33]. However, the exact mechanisms whereby CD4^+^Foxp3^+^ T cells mediate ACAID have however not yet been clarified. Since both CD4^+^ and CD8^+^ T cells expressing Foxp3 play a role in ACAID, it is interesting to find out which Foxp3^+^ T cell population is indispensable or whether both populations are needed for the development of ACAID.

In conclusion, our study revealed an inhibitory effect of CD8^+^ T cells on the expression of the DTH response and an increased expression of Foxp3^+^ on CD8^+^ T cells in an OVA-induced ACAID model. These results suggest that CD8^+^Foxp3^+^ T cells may play a role in the development of ACAID. However, the exact role of this subpopulation of cells in ACAID requires further investigation.
